# Is Osteopontin a Friend or Foe of Cell Apoptosis in Inflammatory Gastrointestinal and Liver Diseases?

**DOI:** 10.3390/ijms19010007

**Published:** 2017-12-21

**Authors:** Tomoya Iida, Kohei Wagatsuma, Daisuke Hirayama, Hiroshi Nakase

**Affiliations:** Department of Gastroenterology and Hepatology, Sapporo Medical University School of Medicine, Minami 1-jo Nishi 16-chome, Chuo-ku, Sapporo 060-8543, Japan; tomoya.iida.0306@gmail.com (T.I.); waga_a05m@yahoo.co.jp (K.W.); d.hirayama@sapmed.ac.jp (D.H.)

**Keywords:** osteopontin, apoptosis, gastrointestinal, liver, inflammation, cacinogenesis

## Abstract

Osteopontin (OPN) is involved in a variety of biological processes, including bone remodeling, innate immunity, acute and chronic inflammation, and cancer. The expression of OPN occurs in various tissues and cells, including intestinal epithelial cells and immune cells such as macrophages, dendritic cells, and T lymphocytes. OPN plays an important role in the efficient development of T helper 1 immune responses and cell survival by inhibiting apoptosis. The association of OPN with apoptosis has been investigated. In this review, we described the role of OPN in inflammatory gastrointestinal and liver diseases, focusing on the association of OPN with apoptosis. OPN changes its association with apoptosis depending on the type of disease and the phase of disease activity, acting as a promoter or a suppressor of inflammation and inflammatory carcinogenesis. It is essential that the roles of OPN in those diseases are elucidated, and treatments based on its mechanism are developed.

## 1. Introduction

Cancer epidemiologists have described three carcinogenesis factors: daily diet, smoking, and inflammation [1]. Although they have estimated that approximately 75% of cancers were due to those factors in 1980s, the percentage decreased to 43% in 2000s [1]. Among these three factors, inflammation is important because it does not involve lifestyle, unlike the other two factors.

Inflammation is a physiological response of the body in the attempt to remove harmful stimuli, including damaged cells, irritants, pathogens, and sterile injuries such as cancer, and to begin the healing process. Myeloid cells, including macrophages and neutrophils, are the first immune cells involved in inflammation and are abundant in the tumor microenvironment [2,3]. Fibroblasts are also closely related to inflammation, and they produce collagen and other extracellular matrix components in the tumor microenvironment, stimulating cancer cell proliferation and angiogenesis. Various cytokines, chemokines, other molecules, including osteopontin (OPN), released from immune cells, and fibroblasts relate to the process of inflammatory carcinogenesis [4,5,6,7].

OPN is an extracellular matrix protein. It was identified in 1985 by Heingard et al., as a sialoprotein derived from bovine bone matrix [8]. OPN was also referred to as secreted phosphoprotein 1 (SPP1) and early T lymphocyte activation-1 (ETA-1). This plurality of names reflects the involvement of OPN in multiple physiological and pathological processes [9,10]. OPN belongs to the small integrin binding ligand *N*-linked glycoprotein (SIBLING) family [11]. Although this protein is produced as an approximately 32-kDa, the molecular mass actually ranges from 45 to 75 kDa because of extensive posttranslational modifications [12]. OPN is involved in a variety of biological processes, including bone remodeling, innate immunity, acute and chronic inflammation, and cancer [13,14]. OPN is expressed as a secreted OPN (sOPN) or an intracellular OPN (iOPN) isoform generated from different OPN translational initiation sites [15]. sOPN is an extracellular protein and relates to various physiologic and pathological events, including immune regulation [16], inflammation [17], tumor progression, and metastasis [18]. On the contrary, iOPN is predominantly present in the cytoplasm and lacks the N-terminal signal sequence of sOPN [15]. The expression of OPN is observed in various tissues and cells, including intestinal epithelial and immune cells such as macrophages, dendritic cells (DCs), and T lymphocytes [19].

OPN plays an important role in efficient development of T helper 1 (Th1) immune responses [20] and cell survival by inhibiting apoptosis [21]. The association of OPN with apoptosis has been elucidated. In this review, we described the role of OPN in inflammatory gastrointestinal (GI) and liver diseases, focusing on its association with apoptosis.

## 2. The Role of OPN in Immune Cells, Focusing on Its Association with Inflammation and Apoptosis

**Figure 1:**
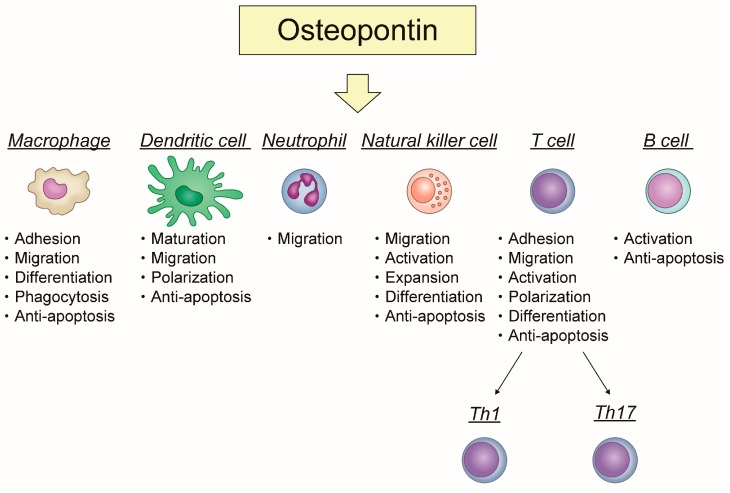
The role of osteopontin in immune cells focusing on the relationship between inflammation and apoptosis.

OPN has specific roles in each immune cell. OPN plays an important role in acute and chronic inflammation. Figure 1 shows how OPN is involved in inflammation and apoptosis related to each immune cell.

### 2.1. Macrophage

Primarily, OPN not only stimulates migration, accumulation, and retention of macrophages at sites of injury but can also modulate their cytokine production by promoting Th1 cell-mediated immunity and stimulating their differentiation from monocytes in both acute and chronic inflammation. OPN controls several immune cell functions, including monocyte adhesion, migration, differentiation, and phagocytosis. OPN has multiple functional adhesive motifs, which allow interactions with various cells, including smooth muscle, endothelial, and inflammatory cells. One of the representative adhesive motifs is the Arg-Gly-Asp (RGD) integrin binding domain. The integrin binding domain of OPN mediates interactions via α_v_β_1_, α_v_β_3_, α_v_β_5_, α_v_β_6_, α_5_β_1_, and α_8_β_1_ integrins [22,23]. In addition, the adjacent Ser-Val-Val-Tyr-Gly-Leu-Arg (SVVYGLR) sequence interacts with α_9_β_1_, α_4_β_1_, and α_4_β_7_ integrins, which are present on the surface of immune cells such as macrophages, T-cells, and neutrophils [24]. The interaction of OPN, not only with α_4_ and α_9_ integrins but also with CD44, influences migration of macrophages. Of note, OPN inhibits macrophage apoptosis by interacting with α_4_ integrin and CD44 [25,26]. Moreover, iOPN increases nuclear factor kappa-light-chain-enhancer of activated B cells (NF-κB) activation through phosphorylation and degradation of nuclear factor of kappa light polypeptide gene enhancer in B-cells inhibitor, α (IκBα) by inducing the IκB kinase α/β (IKKα/β) activity [27,28].

### 2.2. Dendritic Cell (DC)

OPN plays a key role in DC maturation, migration, and polarization [29]. The expression of OPN in immature DCs is higher than in mature DCs. Therefore, OPN works as an autocrine and/or paracrine signal for DC maturation [30]. OPN is involved in the mechanism of DC migration by interacting with α_v_ integrin and CD44 [31]. In addition, OPN acts as a pro-survival signal for DCs, because OPN blocking results in their decreased expression of costimulatory and major histocompatibility complex (MHC) class II molecules, and increased apoptosis [30,31].

### 2.3. Neutrophil

OPN induces neutrophil migration. It is dependent on ERK and P38 MAP kinases activation [32]. OPN seems to affect neutrophil recruitment via integrin-α_v_-dependent suppression of CXC chemokine receptor 2 (CXCR2) internalization in neutrophils [33,34]. No studies demonstrating the association of OPN with apoptosis in neutrophils are available.

### 2.4. Natural Killer (NK) Cell

OPN plays a key role in increasing NK cell migration and activation. iOPN regulates homeostasis and function in NK cells. The expression of iOPN in NK cells is essential for successful navigation through the contraction phase of expansion and generation of long-lived NK cells with increased functionality [35]. As for the association of NK cells with apoptosis, NK cell-induced apoptosis in tubular epithelial cells has been reported as well as the contribution to renal ischemia reperfusion injury [36]. A beneficial role of blocking OPN expression in renal ischemia-reperfusion injury associated with NK cell-mediated downregulation of inflammatory cytokines and chemokines has been reported, demonstrating that the histologic architecture and apoptosis of renal tissue improved in anti-OPN antibody-treated mice [37]. Moreover, deficient expression of iOPN in NK cells causes impaired expansion and increased apoptosis of these cells following stimulation with interleukin 15 (IL-15), resulting in defective immune response to viral infection and tumor [38].

### 2.5. T Cell

OPN is involved in Th cell polarization by enhancing Th1 and Th17 differentiation and inhibiting Th2 cytokine expression. A study reported a relationship between iOPN and T follicular helper (TFH) [39]. OPN is also known as ETA-1 due to its high expression in activated T cells. It has been shown that an anti-OPN antibody promoted apoptosis of activated T cells, particularly CD4+ T cells, by inhibiting activation of NF-κB in a model of rheumatoid arthritis (RA) [40].

### 2.6. B Cell

OPN works as a polyclonal B-cell activator. OPN stimulates immunoglobulin (Ig) production by B cells in vitro. In addition, it was reported that overexpressing OPN induced elevated serum levels of several isotypes of Ig in vivo [41,42]. OPN is related to autoimmune diseases such as RA, systemic lupus erythematosus [43,44], and multiple sclerosis (MS) [45,46]. In an MS mouse model, B-cell activating factor (BAFF) induces B-cell lymphoma 2 (BCL-2) expression in T cells by upregulating OPN secretion from B cells [47].

## 3. The Association of OPN with Apoptosis in Inflammatory GI and Liver Diseases

**Figure 2:**
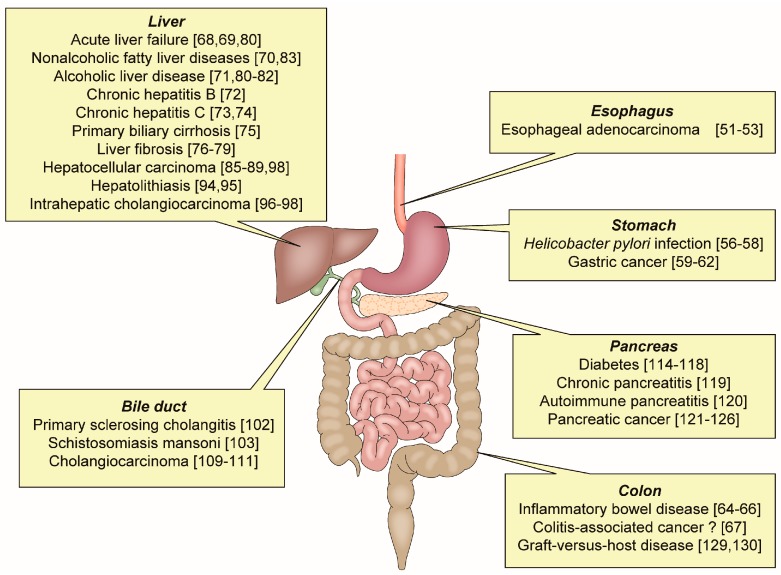
Inflammatory gastrointestinal and liver diseases related to osteopontin.

OPN has an important role in various inflammatory GI and liver diseases (Figure 2).

**Figure 3:**
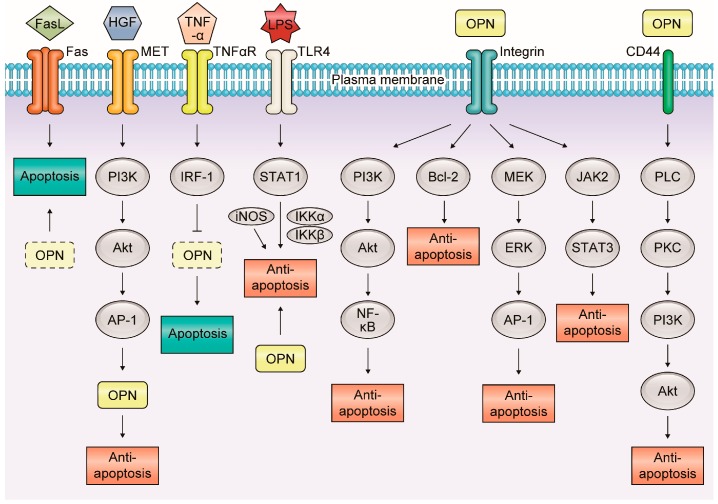
Molecular mechanisms of the relationship between osteopontin and apoptosis in inflammatory gastrointestinal and liver diseases. In inflammatory gastrointestinal and liver diseases, OPN is associated with apoptosis via various molecular mechanisms. OPN mainly plays an anti-apoptotic role in many signaling pathways interacting with each receptor.

Apoptosis is an essential process for maintaining homeostasis in normal tissues and is deeply connected with inflammation and carcinogenesis [48]. In inflammatory GI and liver diseases, OPN is associated with apoptosis via various molecular mechanisms (Figure 3).

### 3.1. Esophageal Adenocarcinoma (EAC)

EAC is a malignant tumor caused by chronic inflammation. Chronic gastroesophageal reflux disease is a major risk factor for the development of Barrett’s esophagus, which could lead to EAC [49,50]. The prognosis of EAC patients with the expression of OPN is poor [51]. The expression of OPN is considerably elevated in EAC compared to Barret’s esophagus and low- or high-grade dysplasia. The same study has demonstrated that all five isoforms of OPN (OPNa, OPNb, OPNc, OPN4, and OPN5) were co-overexpressed in the majority of primary EACs and that individual OPN isoforms showed distinct phenotypes, yet acting collectively in tumor invasion and dissemination in EAC/OPN cell models [52]. In addition, increased expression of multiple genes such as matrix metalloproteinases (MMPs) and OPN in the MET pathway associated with invasive growth was observed in EACs. Treatment of EAC-derived cell lines with geldanamycin, an inhibitor of tyrosine kinases, including MET receptor kinases, reduces cell migration and induces EAC cell apoptosis. These results indicate that MET pathway, which is correlated with OPN, upregulates EAC cell migration and decreases EAC cell apoptosis [53].

### 3.2. Helicobacter pylori Infection and Gastric Cancer (GC)

*H. pylori* is the well-recognized cause of GC and has been classified by World Health Organization as group I carcinogen. *H. pylori*-infected patients develop GC via multistep processes, including chronic gastritis, atrophic gastritis, intestinal metaplasia (IM), dysplasia, and ultimately GC [54,55]. OPN contributes to immune escape of *H. pylori* via inhibition of inducible nitric oxide synthase (iNOS) production by macrophage [56]. A study including 105 *H. pylori*-infected patients has shown that increased expression of gastric OPN during *H. pylori* infection correlated with a more severe gastric inflammation and the presence of IM [57]. A study including *H. pylori*-infected patients with or without IM has also shown that *OPN* polymorphisms predisposed to IM development in *H. pylori*-infected males [58].

Several studies have reported increased expression of OPN in GC. A study has reported that the pro-survival and anti-apoptosis activities of OPN in GC cells were mediated in part through phosphoinositide 3-kinase (PI3K)/AKT pathway via α_v_β_3_ integrins [59]. PI3K/AKT pathway and hypoxia-inducible factor-1 are involved in the tumor-promoting function of OPN, which induces pro-survival and anti-apoptosis signaling in GCs after the survival pathway is activated [60]. In GC, the NF-κB pathway is also crucial for cell survival via initiation of the gene expression of anti-apoptotic factors [61]. In addition, in OPN-knockout (KO) mice treated with *N*-methyl-*N*-nitrosourea and infected with *H. pylori*, the loss of OPN decreased *H. pylori*-induced gastric carcinogenesis by suppressing pro-inflammatory immune response and augmenting signal transducer and activator of transcription 1 (STAT1) and iNOS-mediated apoptosis of gastric epithelial cells [62].

### 3.3. Inflammatory Bowel Disease (IBD) and Colitis-Associated Cancer (CAC)

IBD is a chronic inflammatory disease involving idiopathic inflammation, mainly in the GI tract. It comprises ulcerative colitis (UC) and Crohn’s disease (CD) which cause chronic intestinal inflammation, mucosal damage, and epithelial barrier dysfunction. Various cytokines, including tumor necrosis factor-α (TNF-α), are related to the pathophysiology of IBD. TNF-α is a main therapeutic target for IBD. TNF-α induces the transcription factor interferon regulatory factor-1 (IRF-1) in intestinal epithelial cells in vitro. Induction of IRF-1 is associated with epithelial cell apoptosis by OPN suppression [63]. Previous studies have shown that OPN protected from acute colitis but not from chronic colitis in an experimental colitis model [64,65]. It has also been reported that OPN-KO mice exhibited considerably decreased disease activity compared to wild-type (WT) mice as evidenced by reduced rectal bleeding, weight loss, and histological intestinal injury [66]. These paradoxical data reflect the possibility that the role of OPN may be different depending on the type of disease and the disease phase. It is also possible that the role of OPN differs in epithelium and immune cells.

There is no report showing a direct association of OPN with CAC. IRF-1 is related to OPN and apoptosis in IBD [63]. In dextran sulfate sodium (DSS)-treated mice, more colonic dysplasia was observed in IRF-1-KO mice than in WT mice. In addition, microarray analysis comparing colonic gene expression in IRF-1-KO mice and WT mice revealed decreased expression of caspases and tumor suppressor genes in the IRF-1-KO mice [67]. Further studies are needed to elucidate the possible association of OPN with CACf.

### 3.4. Liver Diseases

In the liver, OPN interacts with integrins, CD44, vimentin, and MyD88 signaling pathway, inducing infiltration and migration of immune cells. OPN is a chemoattractant for macrophages and neutrophils during injury in inflammatory liver diseases. 

OPN is involved in many liver diseases such as acute liver failure (ALF) [68,69], non-alcoholic fatty liver disease (NAFLD) [70], alcoholic liver disease [71], chronic hepatitis B [72], chronic hepatitis C [73,74], primary biliary cirrhosis [75], and liver fibrosis [76,77,78,79]. However, the roles of OPN in such liver diseases are still controversial. While OPN interacts with neutrophil α_4_β_1_ and α_9_β_1_ integrins, contributing to hepatic neutrophil transmigration and activation, leading to further injury in a rat alcoholic steatohepatitis model [80], OPN deficiency does not prevent but promotes alcoholic neutrophilic hepatitis in mice [81]. In addition, a protective role of OPN in liver has been reported. Transgenic expression of OPN in hepatocytes reduces alcohol-induced hepatic steatosis, balloon cell degeneration, lipid peroxidation, inflammation, and plasma alanine aminotransferase (ALT) activity [82]. A few studies have reported the association of OPN with apoptosis in a rat liver disease model. A study has shown that the expression of BCL-2 was downregulated in ALF and NAFLD, indicating that OPN participated in promoting apoptosis [83]. In addition, OPN enhanced inflammation and cell proliferation, attenuated cell apoptosis, and ultimately facilitated liver regeneration at the termination stage of liver regeneration [84].

OPN plays a crucial role in the oncogenesis of hepatocellular carcinoma (HCC), and overexpression of OPN is positively correlated with tumor progression [85]. Another study has also reported that overexpression of OPN led to intrahepatic metastasis, early recurrence, and poorer prognosis of surgically resected HCC [86,87]. There are several studies showing the association of OPN with HCC apoptosis. In HCC cell line, downregulation of OPN suppresses growth and metastasis of HCC by induction of apoptosis. The same study has reported that OPN silencing in HCC cells resulted in suppression of α_v_, β_1_, and β_3_ integrin expression, inhibition of NF-κB signaling activation, and blockade of BCL-2/B-cell lymphoma-extra large (BCL-XL) and X-linked inhibitor of apoptosis protein (XIAP) expression, increase of BAX expression, inducing mitochondria-mediated apoptosis [88]. In OPN-KO mice, hepatic carcinogenesis is considerably inhibited by OPN deficiency, accompanied by the increase of apoptotic cell death. The same study has also shown that OPN was an important factor for inducing c-Jun-mediated epidermal growth factor receptor transcription, resulting in the inhibition of apoptotic cell death [89].

In addition, hepatolithiasis is an important factor of intrahepatic cholangiocarcinoma (ICC) [90,91,92,93]. There are only few studies investigating the association of OPN and hepatolithiasis-related apoptosis [94,95]. Data from 17 hepatic resection specimens with hepatolith have shown positive immunostaining results for OPN in the cytoplasm of the epithelial cells of stone-containing intrahepatic bile ducts and intramural and extramural glands, and in stones. The results have suggested that OPN from the intrahepatic bile ducts and peribiliary glands played a role in the formation of intrahepatic stones [94]. In addition, a study on surgically resected specimens from 73 patients with ICC has shown negative correlation between the expression of OPN and tumor aggressiveness and clinical outcome [96]. On the contrary, another study, using tissue microarray analysis, indicated a positive correlation between the expression of OPN and poor prognosis [97]. There are few reports on the association of OPN and ICC apoptosis. The inhibition of microRNA-21, upregulated in HCC and ICC, reduces liver fibrosis and prevents tumor development by inducing apoptosis of CD24+ progenitor cells [98].

### 3.5. Bile Duct Diseases

The etiology of CC is typically associated with chronic biliary inflammation, which can be observed in primary sclerosing cholangitis (PSC), pancreaticobiliary maljunction, or infection, including hepatitis B virus (HBV) and HCV infections [99,100,101]. There are few reports on the association of OPN with bile duct diseases. In PSC mice with 3, 5-diethoxycarbonyl-1, 4-dihydrocollidine-induced sclerosing cholangitis, the genetic loss of neither OPN nor TNF-α receptor-1 considerably affects the pathogenesis of sclerosing cholangitis, ductular reaction, and biliary fibrosis [102]. With regards to a rare disease, schistosome-induced cholangiocyte proliferation and OPN secretion correlate with fibrosis and portal hypertension in human and murine *Schistosomiasis mansoni* [103]. On the other hand, several molecular mechanisms of CC carcinogenesis have been reported, including the KRAS/RAF/MEK/mitogen-activated protein kinase (MAPK) [104], IL-6/signal transducer and activator of transcription 3 (STAT3) [105,106], transforming growth factor β (TGF-β)/SMAD [107], and TNF-α/Snail pathways [108]. However, only few studies have been conducted to investigate the relationship between OPN and CC [109,110]. A study has reported that, in correlation with the upregulation in CC cells and the tumor stroma, serum levels of OPN were elevated in patients with CC compared to in healthy controls and patients with PSC [111]. There is no report directly investigating the association of OPN with apoptosis in biliary tract diseases. However, CC is related to TGF-β-induced apoptosis [112]. In addition, MCL-1, which is a member of the BCL-2 protein family involved in the regulation of apoptotic cell death, is upregulated in CC cell lines via an IL-6/Janus kinase (JAK)/STAT-dependent pathway [113]. Further studies are needed to investigate the association of OPN with apoptosis in bile duct diseases.

### 3.6. Pancreatic Diseases

OPN is associated with diabetes closely related to insulin and glucagon secretion [114,115]. The expression of OPN is accelerated in vascular smooth muscle cells of rats because of the protein kinase C and hexosamine pathway activation-induced high glucose concentration [114]. Oxidant stress is also involved in the accelerated expression of OPN in vascular smooth muscle cells of rats because of the high glucose concentration [115]. In addition, OPN inhibits cytokine-induced apoptosis via reduction of NO and iNOS levels [116], and stimulates β-cell proliferation [117]. β-cell proliferative and anti-apoptotic roles have been described for glucose-dependent insulinotropic polypeptide, in addition to its action as an incretin hormone [118].

OPN is expressed in acinar cells, ductal cells, and invading macrophages in chronic pancreatitis (CP) specimens but not in normal pancreas [119]. In an autoimmune pancreatitis (AIP) model of WBN/Kob rats, the expression of OPN in centroacinar cells in CP with calcification and in AIP is considerably greater than that in normal pancreas [120]. OPN is also used as a discriminating marker for pancreatic cancer (PC) and CP [121]. OPN influences the invasiveness of PC cells and increases in neoplastic and inflammatory conditions [122]. A meta-analysis has shown that an elevated serum OPN level might be used as a promising diagnostic tool for early identification of PC [123]. In addition, a recent study has reported that high glucose levels accelerated cell proliferation and increased the secretion of OPN in human pancreatic duct epithelial cells [124]. However, another study has reported that the presence of OPN in PC might have a protective effect independent of tumor stage [125], and a recent study has concluded that the relationship between OPN and PC remained unclear [126]. There are no reports which directly show the association of OPN with apoptosis in PC.

### 3.7. Graft-Versus-Host Disease (GVHD)

GVHD is one of the major complications after allogeneic hematopoietic stem cell transplantation (HSCT). Acute GVHD is characterized by the infiltration of donor T lymphocytes that are specific against host antigens and epithelial cell apoptosis [127,128]. A study has indicated that OPN exacerbated GVHD by stimulating CD8+ T cells and that anti-OPN antibody treatment inhibited the development of acute GVHD in a mouse model [129]. In addition, OPN deficiency in donor cells affects the onset of acute Gl GVHD by regulating apoptosis of the intestinal cells via the Fas-Fas ligand pathway [130]. This discrepancy may be explained by differences in models and the observational period after HSCT.

Inflammatory gastrointestinal and liver diseases related to osteopontin-induced apoptosis were summarized in Table 1.

## 4. Conclusions

In this review, we showed the role of OPN in inflammatory GI and liver diseases in association with apoptosis. OPN changes its association with apoptosis depending on the type of disease and the phase of disease activity, and acts as a promoter or a suppressor in inflammation and inflammatory carcinogenesis. It is essential that the roles of OPN in those diseases are elucidated, and treatments based on its mechanism are developed.

## Figures and Tables

**Table 1:** Inflammatory gastrointestinal and liver diseases related to osteopontin-induced apoptosis.

Organ	Disease	Osteopontin-Induced Apoptosis	Reference
Esophagus	Esophageal adenocarcinoma	↓	[52,53]
Stomach	Gastric caner	↓	[59,60,61,62]
Colon	Inflammatory bowel disease	↓	[63]
	Colitis-associated cancer	↓?	[63,67]
	Graft-versus-host disease	↑/↓	[129]/[130]
Liver	Acute liver failure	↑	[83]
	Nonalcoholic fatty liver disease	↑	[83]
	Hepatocellular carcinoma	↓	[88,89]
Bile duct	Cholangiocarcinoma	↓?	[113]

↑: upregulated, ↓: downregulated, ?: possibility.
